# Genetic Polymorphisms of Vitamin D Pathway Predict Antiviral Treatment Outcome in Slow Responder Naïve Patients with Chronic Hepatitis C

**DOI:** 10.1371/journal.pone.0080764

**Published:** 2013-11-14

**Authors:** Edmondo Falleti, Sara Cmet, Carlo Fabris, Giovanna Fattovich, Annarosa Cussigh, Davide Bitetto, Elisa Ceriani, Ilaria Lenisa, Denis Dissegna, Donatella Ieluzzi, Anna Rostello, Mario Pirisi, Pierluigi Toniutto

**Affiliations:** 1 Department of Medicine and Pathology Clinical and Experimental, Medical Liver Transplantation Unit, Internal Medicine, University of Udine, Udine, Italy; 2 Gastroenterology Clinic, Department of Medicine, Azienda Ospedaliero-Universitaria, University of Verona, Verona, Italy; 3 Department of Clinical and Experimental Medicine, Università del Piemonte Orientale “A. Avogadro”, Novara, Italy; 4 Interdepartmental Research Centre for Autoimmune Diseases (IRCAD), Novara, Italy; University of Sydney, Australia

## Abstract

Vitamin D serum levels seem to influence antiviral response in chronic hepatitis C. Vitamin D pathway is controlled by genes presenting functional single nucleotide polymorphisms (SNPs). Data regarding the association between these polymorphisms and the rate of sustained viral response (SVR) following antiviral treatment in chronic hepatitis C virus (HCV) infection are largely incomplete. Aim of this study was to evaluate if the carriage of different SNPs of these genes could influence the rate of SVR in patients treated with interferon plus ribavirin. Two hundred and six HCV positive patients treated with PEG-interferon plus ribavirin were retrospectively evaluated. Polymorphic loci rs7041 G>T and rs4588 C>A of the vitamin D transporter GC-globulin, rs10741657 G>A of the vitamin D 25 hydroxylase CYP2R1 and rs10877012 G>T of vitamin D 1-hydroxylase CYP27B1 were genotyped. A genetic model named VDPFA (vitamin D Pathway Functional Alleles) was constructed considering for each patient the sum (from 0 to 8), derived from every functional allele carried, associated with the achievement of SVR. Three groups were identified: those carrying ≤4 VDPFA (N=108), those carrying 5-6 VDPFA (N=78) and those carrying ≥7 VDPFA (N=20). Significant associations were found between the rates of SVR and the VDPFA value both in all (61/108, 53/78, 17/20, p=0.009) and in 1/4-5 HCV genotypes (17/56, 23/43, 6/8, p=0.003). Moreover in patients who don’t achieve rapid viral response (RVR) SVR and VDPFA were found to be in stronger associations in all (12/55, 17/39, 7/9, p<0.001) and in 1/4-5 HCV genotypes (4/41, 12/31, 5/6, p=0.001). VDPFA value ≥7 could aid to select, among RVR negative difficult to treat 1/4-5 HCV genotypes, those achieving SVR. These observations could permit to extend the indication to adopt dual antiviral therapy beyond RVR positivity rule without reducing the chances of SVR.

## Introduction

With the introduction of hepatitis C virus (HCV) protease inhibitors, antiviral therapy of chronic hepatitis C has been dramatically evolved. Waiting for the incoming third generation pan-genotypic HCV protease and polymerase inhibitors which will be used without interferon and ribavirin [1], the current adopted triple therapy regimens, using boceprevir or telaprevir, with pegylated interferon-α and ribavirin, greatly increased the rate of sustained viral response (SVR) in HCV genotype 1 infected patients. Despite the enhance of SVR rate, triple therapy schedules are complex and are engraved by new and more serious adverse events compared to standard dual therapy. While triple therapy appears to be the only suitable choice in the treatment of patients who previously failed to achieve SVR with standard dual treatment, more questionable is the cost-effectiveness ratio of triple therapy in treating all HCV genotype 1 naïve patients [2]. Thus, despite the progress of triple therapy there is still room for optimizing the efficacy of the less toxic and less costly standard dual antiviral therapy in naïve patients. This aim could be obtained by further refining the identification of the pre and on treatment factors associated with maximal benefit from dual therapy.

Several predictors of successful treatment of chronic hepatitis C have been identified [3]. Factors related to the virus appear to be carefully characterized: infection by HCV genotypes 2 and 3 and a low basal HCV viral load confer a much greater possibility of a successful treatment. Host predictors are classifiable into genetic and non-genetic. Among the latter higher degree of liver inflammation and fibrosis, lower serum cholesterol and higher serum IP-10 and γ-glutamyl-transpeptidase (γ-GT) appear to have a major role [4,5]. Among the former, the major genetic determinant of HCV clearance has been identified in interleukin 28B (IL-28B) polymorphisms. Possessing the IL-28B rs12979860 C/C genotype confers a great advantage for HCV clearance with standard therapy than the carriage of one or two T alleles [6].

Beside the classical action on calcium metabolism, vitamin D appears to possess an important immune-modulator effect [7]. In fact, pre-therapy serum 25(OH) vitamin D levels were found to be an independent predictor of SVR in naïve HCV genotype 1 infected patients treated with standard dual therapy [8]. Basal serum 25(OH) vitamin D levels >20 ng/mL were associated to an increase of about 20% in the probability to achieve SVR in patients carrying the IL-28B C/C genotype [9]. Moreover, vitamin D supplementation was associated with an enhancement of SVR rate in patients with chronic hepatitis C irrespectively of the viral genotype [10,11].

Vitamin D undergoes two activation processes before its interaction, as active 1,25-(OH)_2_ vitamin D, with vitamin D receptor [12]. The first activation is performed in the liver by CYP2R1 and produces the 25-hydroxylated form of vitamin D [13]. This step produces the main circulating, though biologically inactive, 25-OH vitamin D form, which circulates bound to GC-globulin, also named vitamin D binding protein. GC-globulin is a serum α_2_-globulin, synthesized only by the liver in three genetically determined isoforms [14]. By means of CYP27B1, vitamin D is subjected to its second hydroxylation which leads to the 1,25(OH)_2_ active vitamin D form. This activation occurs predominantly in the proximal tubule of the kidney, although, in the last years, it has been also demonstrated in respiratory epithelial cells, in epidermal keratinocytes and more importantly in macrophages and dendritic cells [15]. Single functional nucleotide polymorphisms of genes of CYP2R1, GC and CYP27B1 are known to influence the vitamin D serum levels [16] but it is still unknown if they could have a clinical significance in influencing the success of antiviral therapy of chronic hepatitis C.

This study aimed to assess whether the genetic polymorphisms rs10741657 G>A of CYP2R1, rs7041 G>T and rs4588 C>A of GC-globulin and rs10877012 G>T of CYP27B1, sequentially involved in the vitamin D pathway, could exert a cumulative effect in influencing the achievement of SVR in naïve patients with chronic hepatitis C treated with interferon plus ribavirin dual therapy.

## Materials and Methods

### Ethics Statements

The study was conducted according to the principles of the Declaration of Helsinki and approved by the Internal Review Boards of the Department of Medical Sciences Experimental and Clinical, University of Udine; of the Department of Medicine, University of Verona and of Department of Clinical and Experimental Medicine, University of Novara respectively.

### Patients

The study population included a total of 206 consecutive, treatment-naïve hepatitis C patients of Caucasian ethnicity, who were retrospectively evaluated. The main demographic and clinical characteristics of these patients have been previously reported [14]. All patients received standard dual therapy in three academic centers in northern Italy: Udine (N=71, 34.5%), Verona (N=88, 42.7%) and Novara (N=47, 22.8%)] from September 2005 to October 2009. Chronic hepatitis C infection was defined by the presence of anti-hepatitis C virus antibodies, serum HCV RNA positivity and the persistent elevation of alanine aminotransferase (ALT) for at least six months. In addition, 142 patients had had a liver biopsy performed within the 6 months preceding the start of standard dual therapy. Exclusion criteria were: decompensated liver cirrhosis (Child-Pugh score >6), the presence of hepatocellular carcinoma (HCC), HIV and/or HBV co-infection, autoimmune and/or genetic liver disease (i.e., Wilson’s disease, haemochromatosis), concomitant use of vitamin D or calcium supplementation, active intravenous drug use. The main clinical and demographic characteristics of the studied population are reported in table 1. All the patients signed a written informed consent to participate to the study. An overnight fasting blood sample was drawn to determine the baseline blood tests, including 25-OH vitamin D levels, using DiaSorin 25-OH vitamin D chemo-luminescent immunoassay on a Liaison automatic analyzer, HCV RNA quantification, using real-time PCR (TaqMan, Roche) and HCV genotype, detected using the InnoLipa genotyping kit (Innogenetics). One hundred and nineteen healthy blood donors were used as controls. They were 73 males (61.3%) with a median age of 50 years (range 25-65) and with a median body mass index (BMI) of 24 (range 18-33) Kg/m^2^. No significant difference was observed between patients and controls regarding gender (p=0.084), age (p=0.944) and BMI (p=0.112).

**Table 1:** Demographic and clinical characteristics of the studied population at baseline (N=206).

Age years		48 (40-60)
Female gender		100 (48.5%)
Body mass index kg/m^2^		24 (22-27)
HCV genotypes	1	92 (44.7%)
	2	61 (29.6%)
	3	38 (18.4%)
	4-5	15 (7.3%)
HCV RNA (x 10^3^ IU/mL)		700 (292-1717)
Cholesterol mg/dL		170 (145-194)
ALT IU/mL		68 (41-115)
γ-GT IU/mL		38 (21-72)
HOMA		1.9 (1.0-3.5)
Use of pegylated interferon-α-2a		61 (29.6%)
Grading pre-treatment*		2 (2-4)
Staging pre-treatment*		2 (1-3)
Alcohol consumption g/day		0 (0-10)

Continuous variables are presented as median (inter-quartile range) while categorical variables are presented as frequencies (%). ALT=alanine aminotransferase, γ-GT=gamma-glutamyl transpeptidase, HOMA=homeostasis model assessment, * available in 142 patients

### Molecular biology

Genotyping for the IL-28B rs12979860 C>T, for GC rs7041 T>G (Asp432Glu), for GC rs4588 C>A (Thr436Lys) has been performed as previously described [14]. CYP2R1 rs10741657 G>A and CYP27B1 rs10877012 G>T polymorphisms were performed by polymerase chain reaction-based restriction fragment length polymorphism assay. Specific primers were newly designed. For CYP2R1 rs10741657 G>A a 145 bp amplicon was obtained with the 5’-TGGTGGTTGGGGAGATACTT-3’ forward and 5’-AAGCCATCAGATTGGTGGTAA-3’ reverse primers. For CYP27B1 rs10877012 G>T polymorphism a 187 bp product was obtained with the 5’-GCCTGTAGTGCCTTGAGAGG-3’ forward and 5’-CAGTGGGGAATGAGGGAGTA-3’ reverse primers. The PCR amplifications were carried out in a total volume of 10 µL. Samples containing 10 ng of genomic DNA were subjected to 40 cycles of denaturation (95°C for 30 s), annealing (60°C for 30 s) and elongation (72°C for 30 s) using a Techne TC-5000 thermal cycler. In a total volume of 20 µL, amplified DNA (10 µL) was digested overnight with 2 units of restriction endonucleases using the buffers and temperatures recommended by the manufacturer. MnlI restriction endonuclease was used for CYP2R1 rs10741657 G>A and HinfI for CYP27B1 rs10877012 G>T polymorphisms. The endonucleases were purchased from New England Biolabs, Hitchin, UK. The restricted fragments were 124+21 bp for G allele and 145 bp for A allele of CYP2R1 rs10741657, 138+49 bp for G allele and 187 bp for T allele of CYP27B1 rs10877012. The fragments were resolved by electrophoresis on a 3.5% agarose gel after staining with ethidium bromide.

### Histology

As mentioned above, 142 out of 206 patients (68.9%) underwent a liver biopsy before starting antiviral therapy. Grade and stage were scored according to the Ishak system [17].

### Antiviral therapy schedule and outcomes

All patients were treated with standard dual therapy, consisting in the combination therapy with pegylated interferon-α plus ribavirin. One hundred and forty five patients (70.4%) received pegylated interferon-α-2b (Peg-Intron,™ Schering-Plough, USA) at a dosage of 1.5 μg/kg per week, and sixty one patients (29.6%) received pegylated interferon-α-2a (Pegasys,™ Roche, Basel, Switzerland) at a dosage of 180 μg per week. In patients infected by HCV genotypes 1, 4 and 5, ribavirin (either Rebetol,™ Schering-Plough, USA, or Copegus,™ Roche, Basel, Switzerland) was administered according to body weight (1000 mg/d for patients weighing <75 kg, 1200 mg/d for those weighting ≥75 kg). In the case of infection by genotypes 2 and 3, a flat ribavirin dose of 800 mg/d was used. The duration of standard dual therapy was 48 weeks for genotypes 1, 4 and 5 and 24 weeks for genotypes 2 and 3. The definitions of rapid viral response (RVR), complete early viral response (cEVR), end of treatment viral response (EOT) and SVR were made according to the accepted guidelines [18].

### VDPFA genetic model

Because 25(OH) vitamin D serum concentrations are under genetic control, a genetic model, named VDPFA (Vitamin D Pathway Functional Alleles) was created to assess whether it could influence the rate of SVR obtainment after standard dual therapy. VDPFA was constructed giving a value of 1 to the functional allele of each gene known to be associated with a higher enzymatic efficiency (G allele for CYP2R1 rs10741657 and T allele for CYP27B1 rs10877012) or with higher circulating vitamin D serum concentration (G allele for rs7041 and C allele for rs4588 of GC globulin). A value of 0 was given to the other alleles of the four genetic loci studied. Thus, for each patient a VDPFA value ranging from 0 to 8 was obtained.

### Statistical analysis

Statistical analysis of the data was performed using the BMDP dynamic statistical software package 7.0 (Statistical Solutions, Cork, Ireland). Continuous variables are presented as median (range) and categorical variables as frequencies (%). The associations between categorical variables were evaluated using the Pearson chi-squared test and, when appropriate, the chi-squared test for linear trend. The chi-square G test "Goodness of Fit" was employed to verify whether the proportions of the genetic polymorphisms were distributed in accordance with the Hardy-Weinberg equation. Stepwise logistic regression analysis with a forward approach was performed to identify independent predictors of SVR. To correct the statistical type 1 error of VDPFA genetic model, the rough false discovery rate method was adopted. This method imposed that the genetic polymorphism associations employed in the model must have as significance threshold of 0.026 when evaluated in multiple comparisons of univariate analysis. Linkage disequilibrium for GC rs7041 G>T and GC rs4588 C>A loci was calculated using Haploview software.

## Results

### GC rs7041 G>T, GC rs4588 C>A, CYP2R1 rs10741657 G>A, CYP27B1 rs10877012 G>T polymorphisms

Genotype frequencies of HCV infected patients were not found to significantly differ from what expected from the Hardy-Weinberg equilibrium equation. GC rs7041 G>T and rs4588 C>A polymorphisms, which spans 11 bases from each other on GC globulin gene, were found to be in linkage disequilibrium (r^2^=0.442). Allele frequencies of all polymorphisms were similar in patients and in control subjects, except for the G allele of CYP27B1 which was found to be slightly more frequent in control subjects than in patients (0.798 Vs 0.723, p=0.033).

### Viral response

RVR was achieved by 103 patients (50.0%), cEVR by 150 (72.8%), EOT by 157 (76.2%) and SVR by 131 (63.6%) patients. The association between the achievement of SVR and the main clinical and demographic variables known to influence antiviral response is reported in table 2. In all patients, SVR rate was greatly influenced by HCV genotype, IL-28B rs12979860 C>T polymorphism, baseline cholesterol, γ-GT and 25-OH vitamin D serum levels. In difficult to treat HCV genotypes, besides the prominent role exerted by IL-28B rs12979860 C>T polymorphism, the rate of SVR was influenced by baseline γ-GT, HCV RNA, cholesterol and 25-OH vitamin D serum levels.

**Table 2:** Rates of sustained viral response in chronic hepatitis C virus (HCV) infected patients treated with pegylated interferon-α and ribavirin in relationship to baseline demographic and clinical predictors.

	**All genotypes (N=206)**		**Genotypes 1/4-5 (N=107)**	
	N=131 (63.6%)	p	N=46 (43.0%)	p
Age <50 years	73 (55.7%)	0.823	32 (69.6%)	0.340
Female gender	65 (49.6%)	0.683	20 (43.5%)	0.803
Body mass index <25 Kg/m^2^	84 (64.1%)	0.250	32 (69.6%)	0.340
HOMA index <3	97 (74.0%)	0.185	36 (78.3%)	0.208
Cholesterol >200 mg/dL	36 (27.5%)	0.002	11 (23.9%)	0.024
ALT <60 IU/mL	57 (43.5%)	0.623	22 (47.8%)	0.592
γ-GT <80 IU/mL	114 (87.0%)	<0.001	39 (84.8%)	0.016
HCV RNA <600.000 IU/mL	59 (45.0%)	0.281	27 (58.7%)	0.020
HCV genotype 2-3	85 (64.9%)	<0.001	-	-
Use of pegylated interferon-α-2b	88 (67.2%)	0.182	26 (56.5%)	0.039
Cumulative dose of double therapy >80%	113 (86.3%)	0.021	38 (82.6%)	0.205
Alcohol consumption <20 g/day	115 (87.8%)	0.739	40 (87.0%)	0.806
IL-28B rs12979860 C/C	60 (45.8%)	<0.001	24 (52.2%)	<0.001
25(OH) vitamin D >15 ng/mL	98 (74.8%)	0.026	38(82.6%)	0.009

Data are presented for all and difficult to treat HCV genotypes. The statistical analysis was performed by means of Pearson chi-square test. HOMA=homeostasis model assessment, ALT=alanine aminotransferase, γ-GT=gamma-glutamyl transpeptidase, IL-28B=interleukin 28B

### GC rs7041 G>T, GC rs4588 C>A, CYP2R1 rs10741657 G>A, CYP27B1 rs10877012 G>T polymorphisms and SVR

**Figure 1:**
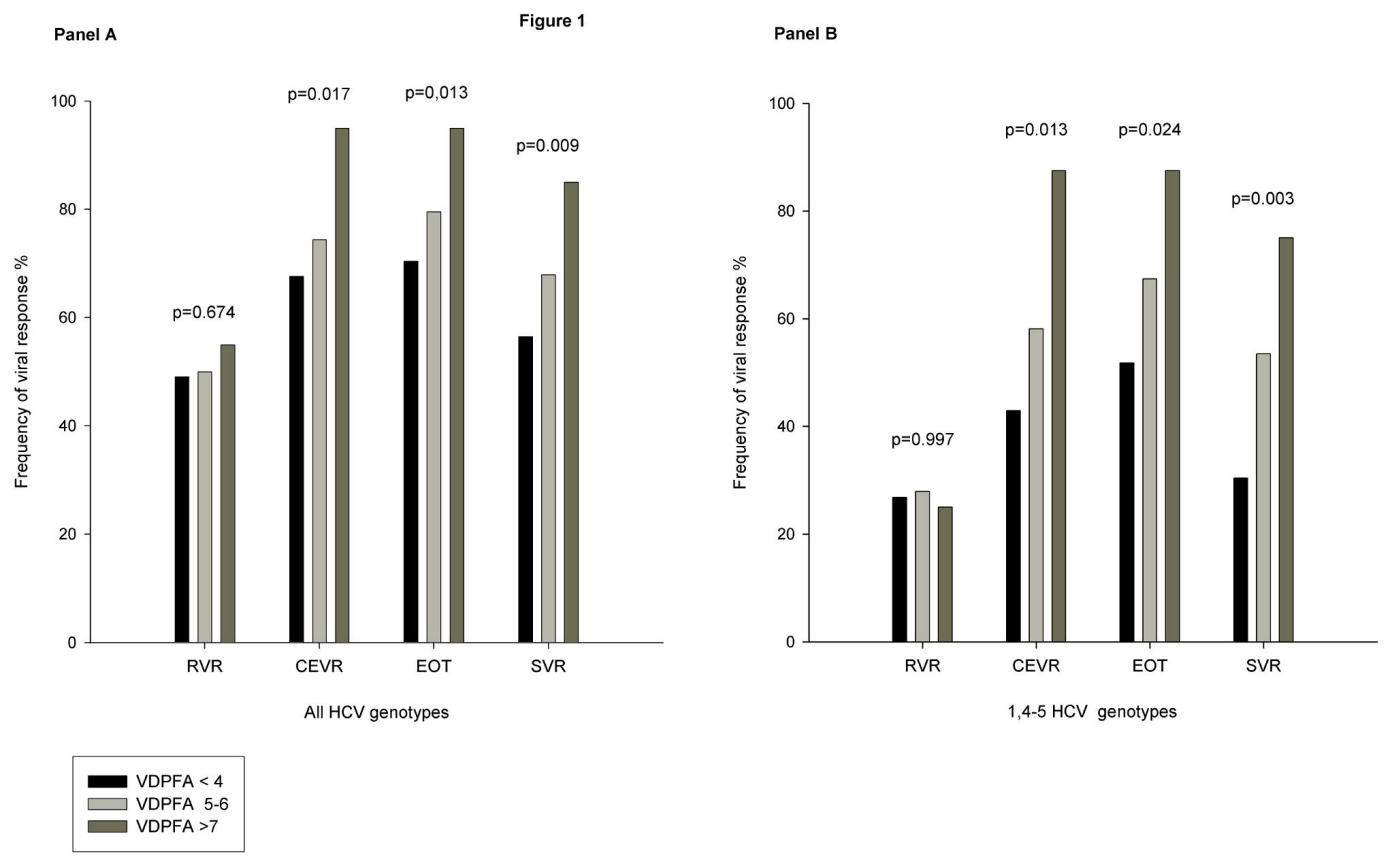
Viral response in relationship with Vitamin D Pathway Functional Alleles (VDPFA). ***PanelA*** Rates of rapid viral response (RVR), complete early viral response (cEVR), end of treatment viral response (EOT) and sustained viral response (SVR) in relationship with the number of Vitamin D Pathway Functional Alleles (VDPFA) carried. Data refer to all patients. Statistical analysis was performed by means of chi-square test for linear trend. ***PanelB*** Rates of rapid viral response (RVR), complete early viral response (cEVR), end of treatment viral response (EOT) and sustained viral response (SVR) in relationship with the number of Vitamin D Pathway Functional Alleles (VDPFA) carried. Data refer to difficult to treat HCV genotypes. Statistical analysis was performed by means of chi-square test for linear trend.

**Figure 2:**
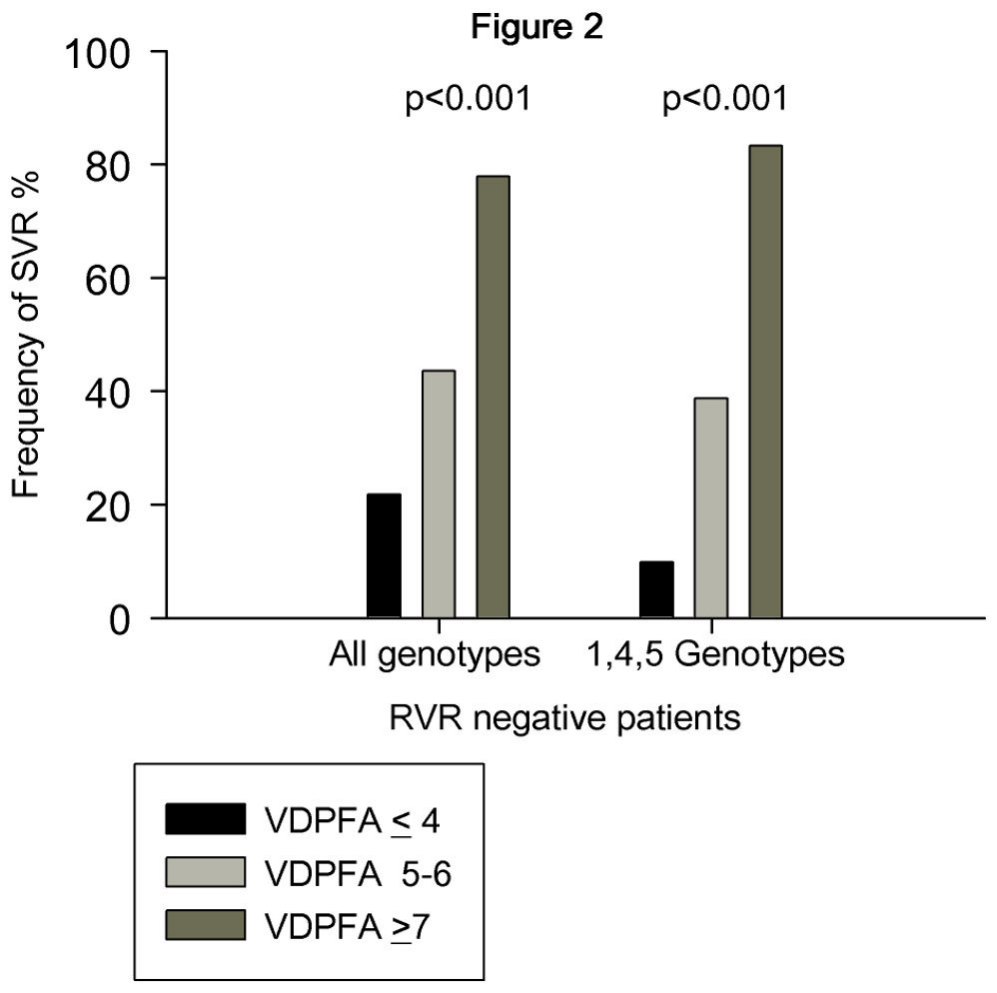
Effect of Vitamin D Pathway Functional Alleles (VDPFA) on antiviral response in RVR negative patients. Rates of sustained viral response (SVR) in patients not achieving rapid viral response (RVR negative) in relationship with the number of Vitamin D Pathway Functional Alleles (VDPFA) carried. Statistical analysis was performed by means of chi-square test for linear trend.

Table 3 shows in all and in difficult to treat 1, 4 and 5 HCV genotypes the associations between the frequencies of the functional allele for the four polymorphisms investigated and the achievement of SVR. The carriage of GC rs7041 G, of GC rs4588 C, of CYP2R1 rs10741657 G and of CYP27B1 T allele was found to be related with SVR achievement, although the relationship did not reach the statistical significance in all but one case. According to the VDPFA genetic model, patients were then stratified in three groups: those carrying ≤4 functional alleles (N=108), those carrying 5-6 functional alleles (N=78) and those carrying ≥7 functional alleles (N=20). A strong significant association was found between the rates of cEVR, EOT, SVR achievement and the number of VDPFA functional alleles carried, either in all (cEVR: 73/108, 58/78, 19/20, p=0.017; EOT: 76/108, 62/78, 19/20, p=0.013; SVR: 61/108, 53/78, 17/20, p=0.009; Figure 1, panel A) and in difficult to treat HCV genotype (cEVR: 24/56, 25/43, 7/8, p=0.013; EOT: 29/56, 29/43, 7/8, p=0.025; SVR: 17/56, 23/43, 6/8, p=0.003; Figure 1, panel B). More interestingly, in patients who don’t achieve RVR a strong association was observed between SVR and VDPFA genetic value, either in all (12/55, 17/39, 7/9, p<0.001) and in difficult to treat HCV genotypes (4/41, 12/31, 5/6, p=0.001, Figure 2).

**Table 3:** Rates of sustained viral response (SVR) in relationship with GC rs7041 G>T, GC rs4588 C>A, CYP2R1 rs10741657 G>A, CYP27B1 rs10877012 G>T polymorphic loci allele frequencies.

		**All genotypes (N=206)**					**Genotypes 1/4-5 (N=107)**				
**Locus**	**Allele**	**SVR+**	**SVR-**	**O.R.**	**95% C.I.**	**p**	**SVR+**	**SVR-**	**O.R.**	**95% C.I.**	**p**
		N=131	N=75				N=46	N=61			
GC rs7041 G>T	G	0.561	0.540	1.089	0.713-1.663	0.679	0.565	0.549	1.067	0.596-1.910	0.815
GC rs4588 C>A	C	0.741	0.707	1.184	0.739-1.897	0.458	0.826	0.713	1.911	0.936-3.934	0.055
CYP2R1 rs10741657 G>A	G	0.733	0.607	1.778	1.135-2.788	0.008	0.707	0.607	1.562	0.843-2.900	0.129
CYP27B1 rs10877012 G>T	T	0.290	0.253	1.204	0.746-1.947	0.422	0.304	0.238	1.403	0.730-2.968	0.275

Data are presented considering all patients and difficult to treat HCV genotypes. The statistical analysis was performed by means of Pearson chi-square test. O.R.=odds ratio, C.I.=confidence interval

### VDPFA genetic model and clinical and demographic variables

A significant association was observed between low 25(OH) vitamin D serum levels (≤15 ng/mL) and a VDPFA value ≤5 in all patients (52/147 Vs 11/59; p=0.018) while it tended to be significant in difficult to treat HCV genotypes (29/79 Vs 4/28; p=0.027). No association was detected between VDPFA values and the main clinical and demographic variables included in table 2. In particular, no relationship was detected at all, between VDPFA value ≤5 and IL-28B rs12979860 T/* polymorphism (95/147 Vs 35/59; p=0.48).

### Multivariate analysis

Stepwise logistic regression analysis was performed to assess whether a VDPFA value (>5 Vs ≤5) could be considered an independent predictor of SVR, in all and in difficult to treat HCV genotypes (table 4). Besides the main known predictors of SVR (HCV genotype, IL28B polymorphism and basal HCV RNA levels), a VDPFA value >5 was found to be independently associated with SVR in all (O.R. 2.30, 95% C.I. 1.02-5.22, p=0.040) and in difficult to treat (O.R. 3.19, 95% C.I. 1.08-9.40, p=0.038) HCV genotypes. Table 5 shows in all and in difficult to treat HCV genotypes who don’t achieve RVR the independent predictors of achieving SVR. In difficult to treat HCV genotypes VDPFA score (>5 Vs ≤5) was found to be an independent predictor of SVR (O.R. 4.48, C.I. 95%: 1.19-16.8; p=0.026) as well as serum γ−GT, serum cholesterol levels and the use of pegylated interferon alfa 2b.

**Table 4:** Stepwise logistic regression analysis in identifying the independent predictors of sustained viral response.

		**All HCV genotypes**			**HCV genotypes 1/4-5**		
		O.R.	95% C.I.	p	O.R.	95% C.I.	p
HCV genotypes: 2-3 Vs 1/4-5		8.51	3.93-18.4	<0.001	-	-	-
IL-28B genotype: C/C Vs T/*		2.51	1.14-5.53	0.009	10.3	3.22-32.8	<0.001
HCV-RNA kIU/mL: ≤600 Vs >600		2.80	1.30-6.04	0.019	5.23	1.81-15.1	0.001
VDPFA: >5 Vs ≤5		2.30	1.02-5.22	0.040	3.19	1.08-9.40	0.038
Cholesterol mg/dL: >200 Vs ≤200		4.01	1.39-11.6	0.018	4.66	1.13-19.1	0.039
Serum γ-GT IU/mL: ≤80 Vs >80		3.31	1.42-7.71	0.001	3.42	1.06-11.1	0.030
25(OH) vitamin D ng/mL: >15 Vs ≤15		2.42	1.05-5.59	0.019	-	-	-

Data are presented considering all patients and difficult to treat HCV genotypes. The variables included in the analysis were those reported in table 2 plus the VDPFA genetic model (>5 Vs ≤5 functional alleles). O.R.=odds ratio, C.I.=confidence interval, IL-28B=interleukin 28B, VDPFA: Vitamin D Pathway Functional Alleles (G for GC rs7041, C for GC rs4588, G for CYP2R1 rs10741657 and T for CYP27B1 rs10877012 loci), PEG-IFN: pegylated interferon; γ-GT=gamma-glutamyl transpeptidase.

**Table 5:** Stepwise logistic regression analysis in identifying the independent predictors of sustained viral response in patients not achieving rapid viral response.

	**All HCV genotypes**			**HCV genotypes 1/4-5**			
	O.R.	95% C.I.	p	O.R.		95% C.I.	p
HCV genotypes: 2-3 Vs 1/4-5	5.90	1.75-19.9	0.003		-	-	-
VDPFA: >5 Vs ≤5	4.54	1.35-15.3	0.018		4.48	1.19-16.8	0.026
Serum γ-GT IU/mL: ≤80 Vs >80	6.89	1.47-32.2	0.012		6.54	1.15-37.1	0.018
PEG-IFN-α-2b Vs PEG-IFN α-2a	4.56	1.42-14.6	0.030		4.51	1.25-16.3	0.025
HOMA index < 3 Vs ≥3	3.80	1.01-14.3	0.008		-	-	-
Cholesterol mg/dL: >200 Vs ≤200	-	-	-		5.46	1.09-27.5	0.034

The variables included in the analysis were those reported in table 2 plus the VDPFA genetic model (>5 Vs ≤5 functional alleles). O.R.=odds ratio, C.I.=confidence interval, IL-28B=interleukin 28B, VDPFA: Vitamin D Pathway Functional Alleles (G for GC rs7041, C for GC rs4588, G for CYP2R1 rs10741657 and T for CYP27B1 rs10877012 loci), PEG-IFN: pegylated interferon; γ-GT=gamma-glutamyl transpeptidase.

## Discussion

This study dealt with genes and their functional polymorphic loci known to be able to influence the serum vitamin D levels: rs10741657 G>A of CYP2R1, rs7041 G>T and rs4588 C>A of GC-globulin and rs10877012 G>T of CYP27B1. The allele frequencies of the gene polymorphic loci studied were similar in patients with chronic hepatitis C and controls; the only exception was represented by the G allele of CYP27B1 rs10877012 G>T found to be slightly more frequent in control subjects than in patients. It must be pointed out that in our patients the allele frequencies were very similar to those detected by Lange et al. in a European cohort of patients with chronic hepatitis C [19,20].

Genome wide association, meta-analysis and large population studies have proved that some genetic determinants are able to predict 25(OH) vitamin D serum concentrations [16,21,22]. Major contribution to serum 25(OH) vitamin D variations appears to be related to GC-globulin gene common genetic polymorphisms. GC rs7041 G>T and rs4588 C>A polymorphisms generate three genetically determined isoforms of this globulin: GC-1s (rs7041G/rs4588C), GC-1f (rs7041T/rs4588C) and GC-2 (rs7041T/rs4588A). The isoforms differ in their amino-acid sequences in position 432 (Glu>Asp) and 436 (Thr>Lys); these modifications produce changes in serum GC-globulin levels in turn associated with variations in serum 25(OH) vitamin D values [16]. Therefore, although appearing in linkage disequilibrium, both polymorphisms were considered in this study. CYP2R1 is expressed only in the liver and is highly conserved among species; it is active in the 25-hydroxylation of both vitamin D_2_ and D_3_. CYP2R1 rs10741657 G>A polymorphism, located 2 kb upstream from the gene, is one of the main polymorphisms found to be associated with 25(OH) vitamin D circulating levels [21,23]. CYP27B1 gene, located on chromosome 11q13.1, is differently regulated in various tissues. In kidney CYP27B1 expression is up-regulated by parathyroid hormone and down-regulated by FGF-23; in contrast, in inflammatory cells, this hydroxylase expression-activity has been found to be regulated by cytokines such as interferon-γ and interleukin-15 [15]. CYP27B1 rs10877012 G>T is a functional polymorphism located in the promoter region of this 1α-hydroxylase. Although a genome wide association study did not detect a relationship between this polymorphic locus and 25(OH) vitamin D levels, in a larger study a strong association was found [22]; furthermore it has been shown that the GG wild type of rs10877012 locus impairs the expression of this enzyme [24]. Finally, Lange et al. demonstrated that this polymorphism could influence serum levels of 1,25(OH)_2_ vitamin D in HCV positive patients [19].

Twin and family studies have reported that 25(OH) vitamin D levels are moderate to high (from 20 to 80%) hereditable [25]. These observations support the idea to construct a genetic model starting from the known functional polymorphisms of genes involved in vitamin D pathway. In effect, a simply genetic model was firstly proposed by Signorello et al. to predict serum 25(OH) vitamin D concentrations [26]. This Author combined the carriage of risk alleles of some GC-globulin and CYP27B1 gene polymorphisms. An inverse relationship was observed between the sum of risk alleles carried and the serum levels of vitamin D. Starting from this finding, it could be reasonable to assess whether, besides basal vitamin D serum levels, a relationship might exist between the VDPFA value and the rate of SVR obtainment with standard dual therapy in patients with HCV chronic infection.

To construct this genetic model, the four polymorphisms studied were singularly associated with SVR obtainment: a positive odd ratio was detected for the major G and C alleles of GC-globulin, the major G allele of CYP2R1 and the minor T allele of CYP27B1. These functional alleles were therefore considered to be potentially implicated in favoring the SVR achievement. Singularly, each polymorphic locus appeared to be weakly related to SVR, with the exception of CYP2R1 rs10741657 when evaluated in all patients. Nevertheless, stratifying the patients for the VDPFA score, the rate of SVR was strongly associated with this index, in all patients and separately in those infected by HCV 1/4-5 genotypes. Taking into account the latter group, patients having a VDPFA value ≤4 achieved SVR in 30.4% of the cases, those having a VDPFA value between 5-6 in 53.3% and finally those having a VDPFA value ≥7 in 75.0%. Interestingly, in patients who did not achieve RVR, VDPFA scores were able to predict more strongly SVR obtainment (9.8%, 38.7% and 83.3% for VDPFA value ≤4, between 5-6 and ≥7 respectively). No association at all was observed between the VDPFA value and the rate of RVR. Finally, VDPFA value was found to be related with serum 25(OH) vitamin D levels, whilst it was unrelated with the main clinical and demographic variables pertaining to patients affected by chronic hepatitis C.

Our results are in agreement with those reported by Lange et al. who demonstrated that CYP27B1 rs10877012 polymorphism, incorporated into our VDPFA model, was associated with the rate of SVR in patients infected by HCV genotypes 1, 2 and 3 [19]. Interestingly in a second study this author observed an influence of this polymorphism in predicting SVR in patients carrying the IL-28B rs12979860 C/T and T/T genotypes [20]. It must be pointed out that in the present study the VDPFA model was found to be a predictor of SVR independently from the main IL-28B rs12979860 polymorphism. VDPFA involves polymorphisms of genes implied either in furnishing 25(OH) vitamin D and in the intra-cellular activation from 25(OH) to 1,25(OH)_2_ vitamin D. It may be suggested that the availability of the precursor and the degree of the local bio-activation are both relevant for the final efficacious effect of vitamin D. Finally, at variance to what occurs for basal vitamin D, VDPFA strongly associates with SVR attainment but not with the achievement of RVR. In this regard, it should be emphasized that basal vitamin D levels may predict the early phases of antiviral treatment, while this effect could subsequently decline [9]. On the contrary, one may argue that genetic polymorphisms effects must be considered constant over time. It may be postulated that the capacity of induce a positive response to antiviral therapy in chronic HCV infection could be influenced by the carriage of genetic variants of genes involved in vitamin D metabolic pathway, in turn conditioning vitamin D bioavailability.

This study has two main limitations: the design was retrospective and it analyzed only Caucasian subjects of North-Italy. Thus a validation prospective cohort of patients should be analyzed to confirm these preliminary results. Moreover, these results would be replicated in patients of other ethnicities carrying different allelic frequencies of the four polymorphic loci evaluated. However, on a clinical point of view, having a VDPFA value ≥7 could aid to select, among naïve difficult to treat HCV genotypes, a sub-group of RVR negative patients still candidates for successfully remaining in standard dual therapy without reducing the rates of SVR achievement.
